# Supplementary material for: Time-lagged Ordered Lasso for network inference
Source: BMC Bioinformatics. 2018 Dec 29;19:545. doi: 10.1186/s12859-018-2558-7 (PMC6311035; doi:10.1186/s12859-018-2558-7)
Supplement: Supplementary file 1 — Supplementary information. (PDF 289 kb) [file 12859_2018_2558_MOESM1_ESM.pdf]

Supplementary Information: Time-lagged Ordered Lasso for network inference

Phan Nguyen and Rosemary Braun

December 3, 2018

S-1 Time-course data and models

Despite the fact that gene expression regulation and other biological functions are dynamic, many existing approaches for GRN reconstruction attempt to predict novel regulatory connections by using static data that is assumed to be measured under steady-state conditions. Given the current advances in high-throughput sequencing techniques, time-course data can be measured at many time points and provide a more comprehensive picture of the dynamic landscape of gene regulation and its resulting biological processes. In particular, time-course data can be used to identify activated genes and changes in differential expression, detect periodicity in gene expression, infer causality between genes, and provide insight into other temporal aspects and mechanisms that cannot be ascertained from static data. However, appropriate modeling assumptions and method modifications are required to effectively leverage the properties that are unique to temporal data. For example, straightforward applications of many existing GRN reconstruction methods to time-course data will treat expression values at different time points as independent samples and disregard the one-way temporal ordering of the data.

One common approach that has been used to analyze the time-course expression data is to assume that it can be modeled with vector autoregression. More specifically, the expression of a gene at a time point is assumed to be linearly dependent on the past expression values of its regulators. For simplicity and computational feasibility, most methods assume that this dependency is short-lived, typically lasting only through the immediately preceding time point, so that the expression of a gene can be described by

$$x_i(t) = \sum_{j=1}^{p} w_{ji} x_j(t - \Delta t), \tag{S-1}$$

where $x_i(t)$ is the expression of gene i at time t, and w_{ji} are weights indicating how much the expression level of gene j influences that of gene i over a time interval Δt. However, depending on factors such as the temporal resolution of the data and delay of influence, the expression of a gene may depend on the expression of its regulators at more than one

preceding time point, in which case the the expression of a gene can now described by

$$x_i(t) = \sum_{j=1}^{p} \sum_{k=1}^{l_{\max}} w_{ji,k} x_j(t - k\Delta t), \tag{S-2}$$

where $x_j(t - k\Delta t)$ is the expression of gene j at the kth previous time point, $w_{ji,k}$ measures how much the expression of gene j at the kth previous time point influences that of gene i at the current time point, and $l_{\max}$ is the model order or maximum lag of a regulator's expression that the expression of gene i may depend on. $\{x_j(t - k\Delta t)\}_{k=1,\ldots,l_{\max}}$ are called the lagged features or variables of gene j.

Differential equations have also been commonly used to model gene regulatory dynamics. With linear ordinary differential equations, the expression of a gene i can be described by

$$\frac{dx_i(t)}{dt} = \sum_{j=1}^{p} a_{ji} x_j(t),$$

where a_{ji} is the rate of influence of the expression of gene j on that of gene i. In order to account for dependence on multiple lags, delay differential equations may be used instead. Using a linear delay differential equation with constant, discrete delays, the expression of a gene i can now described by

$$\frac{dx_i(t)}{dt} = \sum_{j=1}^{p} \sum_{k=1}^{l_{\max}} a_{ji,k} x_j(t - k\Delta t),$$

where $a_{ji,k}$ is the rate of influence of the expression of gene j at the kth previous time point on that of gene i at the current time point. Since microarray data can only collected at discrete time points, discretizing this model results in

$$\Delta x_i(t + \Delta t) = \sum_{j=1}^{p} \sum_{k=1}^{l_{\max}} w_{ji,k} x_j(t - k\Delta t), \tag{S-3}$$

where $w_{ji,k} = a_{ji,k}\Delta t$ and $\Delta x_i(t + \Delta t) = x_i(t + \Delta t) - x_i(t)$.

S-2 Regularization parameter selection

In both method variations, the regularization parameters need to be specified. In the *de novo* case, the true GRN is not known *a priori*, but general trends and heuristics may be useful in choosing these values. One important consideration is the tradeoff between the true and false positive rates as a function of λ. Larger values of λ will promote more regularization and force many of the model coefficients to zero, and applying feature selection will produce fewer predicted edges. Therefore, false positive rates will decrease with large λ, but the true positive rates may decrease as well. Similarly, smaller values of λ will repress regularization and produce more non-zero coefficients and, consequently, more predicted edges. Therefore, true positive rates will increase with small λ, but false positive rates may also increase. In

addition to statistical measures of performance, we may appeal to model selection techniques that automatically choose λ based on the fit of a model to the expression data. Common approaches include cross-validation to simulate out-of-sample performance and information criteria such as AIC and BIC scores to assess model quality and overfitting.

Similar principles may be applied in the semi-supervised case. In this case, we can compute true and false positive rates with respect to the input GRN. However, because the GRN is generally only partially known, these rates should only be interpreted as recovery rates of the input GRN. For smaller values of λ_{edge}, more prior edges will be recovered by the method, and fewer anomalous edges will be proposed. Therefore, λ_{edge} is a reflection of our confidence in the prior information about a network, with smaller values corresponding to a greater certainty that the known edges are true regulatory dependencies. Similarly, for larger values of $\lambda_{\text{non-edge}}$, fewer prior non-edges will be predicted as edges. $\lambda_{\text{non-edge}}$ therefore reflects apprehension to the possibility of novel edges in the network, with larger values corresponding to larger levels of doubt.

S-3 Additional repressilator AUCs

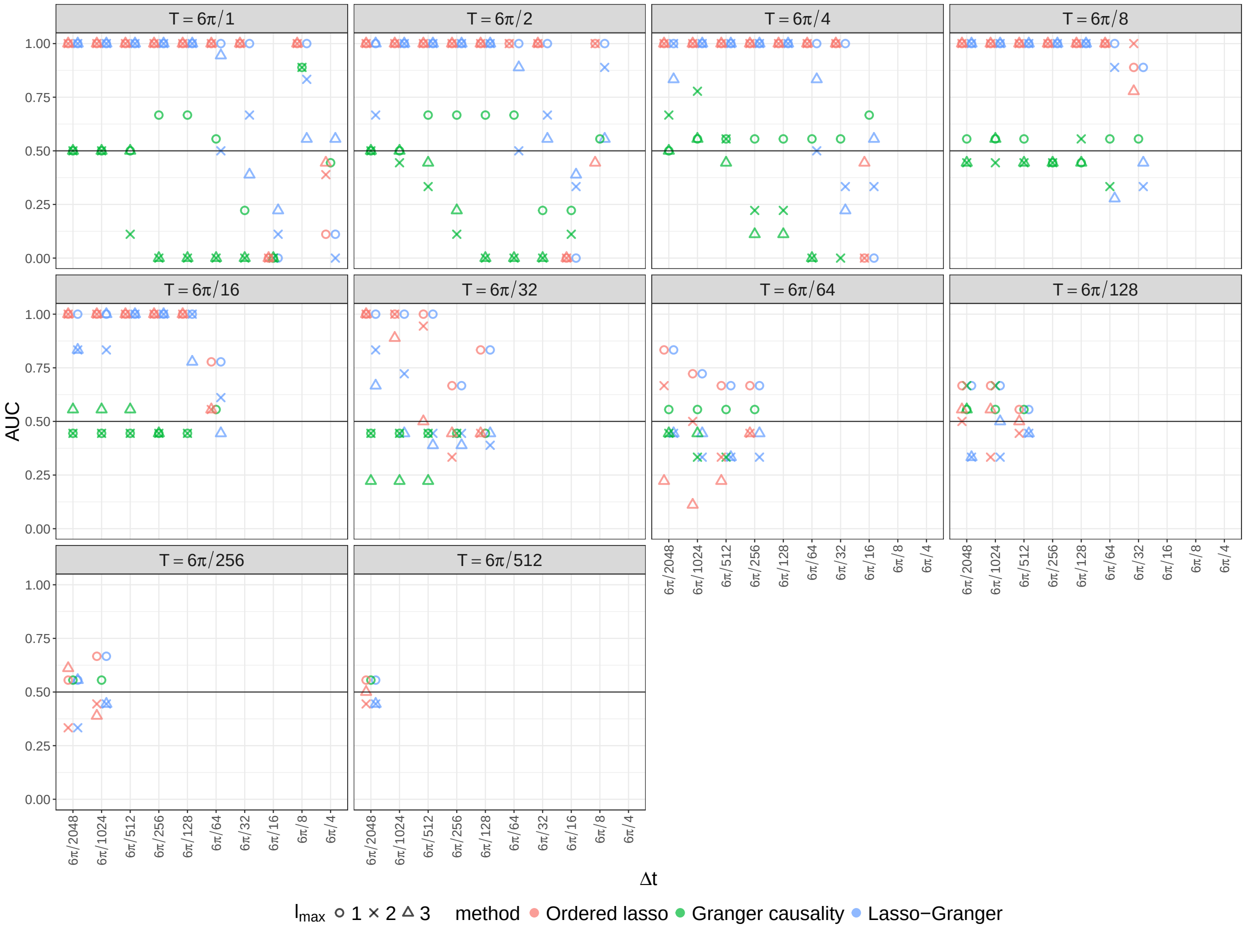

Figure S-1: AUCs for the Ordered Lasso, Granger causality, and Lasso-Granger methods at different model orders $l_{\max}$ when applied to generated expression data for a repressilator. Time series expression data are generated for different periods of time T and sampling rates Δt.

S-4 DREAM network-level analysis

In Figure S-2, the AUCs for each combination of network, method, and model order $l_{\max} \in \{1, \ldots, 4\}$ are shown. At the scale of individual networks, a variety of AUC patterns can be observed that are largely inconsistent across all of the networks. For example, there is no prevalent relationship between the AUCs and $l_{\max}$ for each method. However, the AUCs do appear to be fairly robust to the choice of $l_{\max}$ for each method-network combination and also suggest that all methods are capable of predicting edges correctly at rates better than chance. In addition, it is easy to identify networks in which one method attains the highest AUCs for certain values of $l_{\max}$ and the worst AUCs at other values, while in other networks, the same method may dominate or be dominated by the other methods at all evaluated $l_{\max}$.

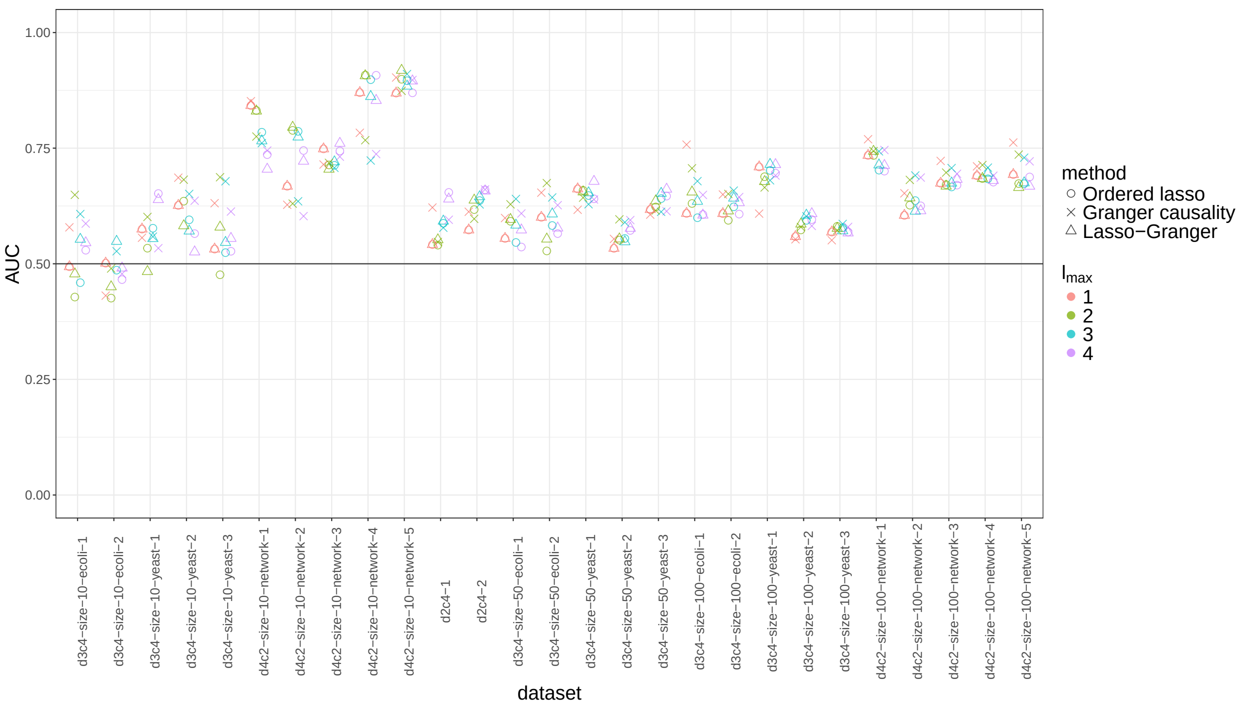

Figure S-2: AUCs for the Ordered Lasso, Granger causality, and Lasso-Granger methods at different model orders $l_{\max}$ when applied to the DREAM datasets.

However, we do note that Granger causality appears to perform marginally better than the other methods on some of the DREAM networks, regardless of the choice of $l_{\max}$.

Conditioning across the different challenges, network sizes, and other factors reveals other elements that may impact the prediction accuracy. In Figure S-3, the AUC differences for Granger causality with respect to the Ordered Lasso are shown. For the DREAM4 challenge datasets, network size appears to influence whether the Ordered Lasso or Granger causality will predict a more accurate network. More specifically, Granger causality attains higher AUCs than the Ordered Lasso does on the 100-node networks, regardless of the model order chosen. In contrast, on the 10-node networks, the Ordered Lasso generally performs better, and for some of these networks, larger differences in accuracy can be observed than in the 100-node networks. When considering the DREAM3 challenge datasets, the type of organism also appears to have an effect on prediction accuracy. For most of the 10-node DREAM3 networks, Granger causality tends to outperform the Ordered Lasso, regardless of organism type. However, based on the AUC differences of the 50- and 100-node networks, the Ordered Lasso tends to perform better than Granger causality on the yeast-based networks, whereas Granger causality tends to do better on the *E. coli*-based networks. This behavior may be due to the differences in the regulatory dynamics of gene expression between prokaryotic and eukaryotic organisms as well the structural differences in their networks. These observations therefore suggest that when attempting to reconstruct networks, no method may perform best on every network, and a preferred method may be dictated by a combination of factors, including network size and type of organism.

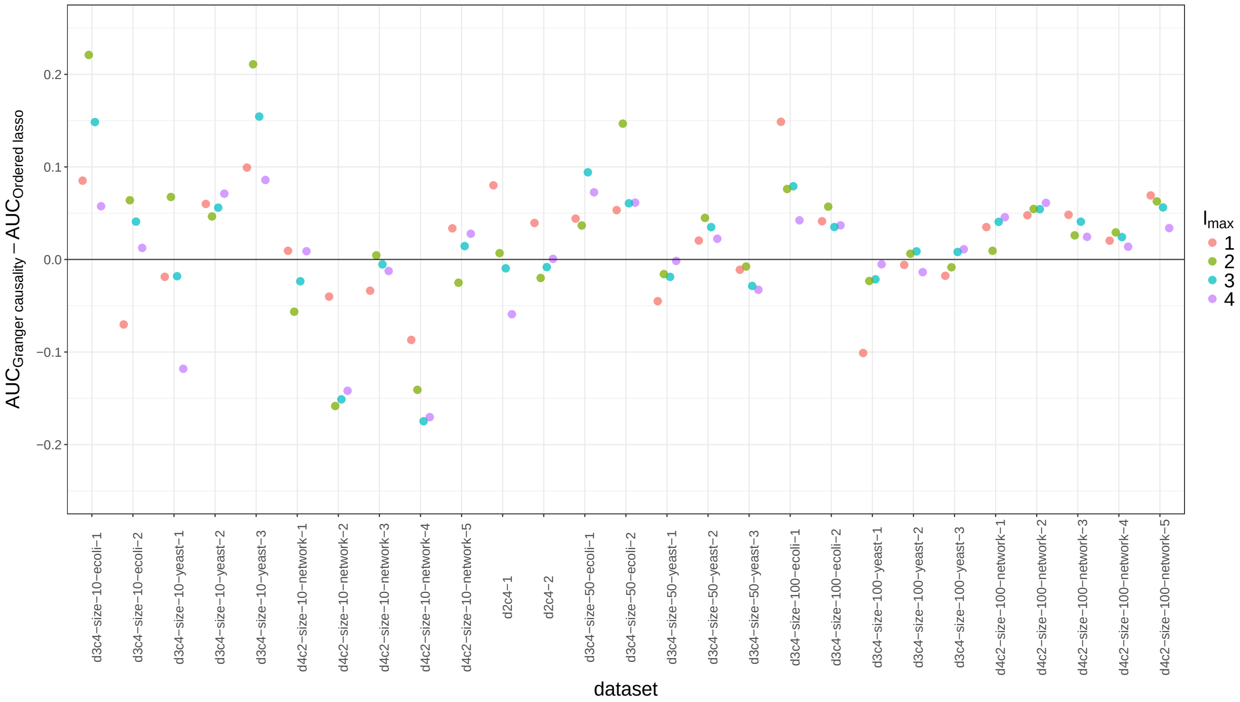

Figure S-3: AUC differences for Granger causality with respect to the Ordered Lasso at different model orders $l_{\max}$ for the DREAM datasets.

S-5 DREAM Granger causality and cross-correlation

In Sections 3.2 and S-4, we observed that Granger causality tended to perform better than the Ordered Lasso did on many of the DREAM networks. One possible reason for this is that many pairs of genes that correspond to true edges may be exhibiting high cross-correlations in absolute value. For the networks in which there is a clear distinction between the distribution of edge and non-edge absolute cross-correlations, the lagged variables of a regulator of a target gene are able to strongly account for much of the evolution in expression of that gene. Recall that bivariate or pairwise Granger causality fits two autoregression models of order $l_{\max}$,

$$y_t = \sum_{i=1}^{l_{\max}} a_i y_{t-i} \tag{S-4}$$

$$y_t = \sum_{i=1}^{l_{\max}} a_i y_{t-i} + \sum_{j=1}^{l_{\max}} b_j x_{t-j}, \tag{S-5}$$

and compares the restricted model (Equation S-4) to the unrestricted model (Equation S-5) using an F-test to assess the explanatory gain of using the lagged values of x in predicting y. In this context, the inclusion of the higher-order lags of a regulator to a target's unrestricted model will therefore tend to result in strong improvements over the restricted model due to the high absolute cross-correlations of the true edges.

In Figure S-4, densities are shown for the absolute values of the edge and non-edge

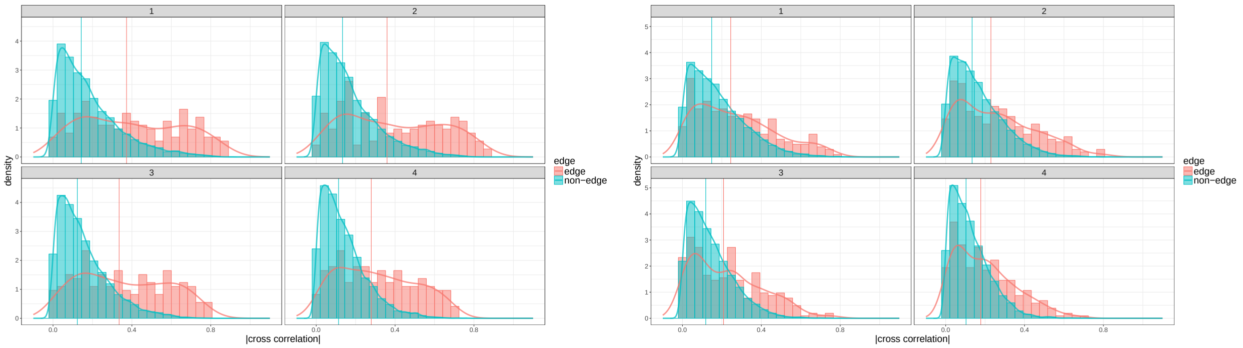

(a) d4c2-size-100-network-1

(b) d4c2-size-100-network-2

Figure S-4: Absolute cross-correlation densities by edge existence for various lags in several DREAM4 challenge networks. Vertical lines indicate empirical medians.

cross-correlations at various lags in several of the DREAM4 challenge networks in which Granger causality outperformed the Ordered Lasso. Overall, we see that edges tend to have higher absolute cross-correlation values than the non-edges do in these networks. As a result, the Granger causality AUCs should be fairly high for many of the DREAM networks. Furthermore, the absolute cross-correlations tend to be higher for the edges than for the non-edges across the different lags, which may explain some of the disparity between the Granger causality and Ordered Lasso AUCs; if the delay in influence of a regulator on a target is longer than one time point and therefore incompatible with the monotonicity constraint of the Ordered Lasso, then the Granger causality AUCs may end up being higher than the Ordered Lasso AUCs.

S-6 Model choices: dependent variables and inclusion of loops

In the main text, we assumed that the dynamics of gene expression can be modeled with Equation S-2, i.e., the expression of a gene is a linear function of the expression of its regulators at multiple preceding time points. In addition, we assumed that the expression model of a gene also included the lagged expression of the gene itself as covariates (loops), even if self-regulation was not evident; in certain cases, this may be a reasonable modeling choice, such as when the expression at a time point can be viewed as the result of the collection of perturbations from its regulators to its expression at previous time points. Since such modeling decisions may appear arbitary and other choices may potentially produce a more accurate network, we now analyze the differences in *de novo* reconstruction accuracy when using the change in expression (Equation S-3) rather than expression (Equation S-2) of a gene at a time point as the output variable to model the gene regulatory dynamics. In addition, we consider the effect of excluding loops from the gene expression models.

In Figure S-5, densities are fit to the Ordered Lasso-based DREAM AUCs and shown for each combination of model output, loop existence, and model order $l_{\max} \in \{1, \ldots, 4\}$, and in Table S-1, p-values are shown for Wilcoxon tests comparing the AUCs with the 0.5-level.

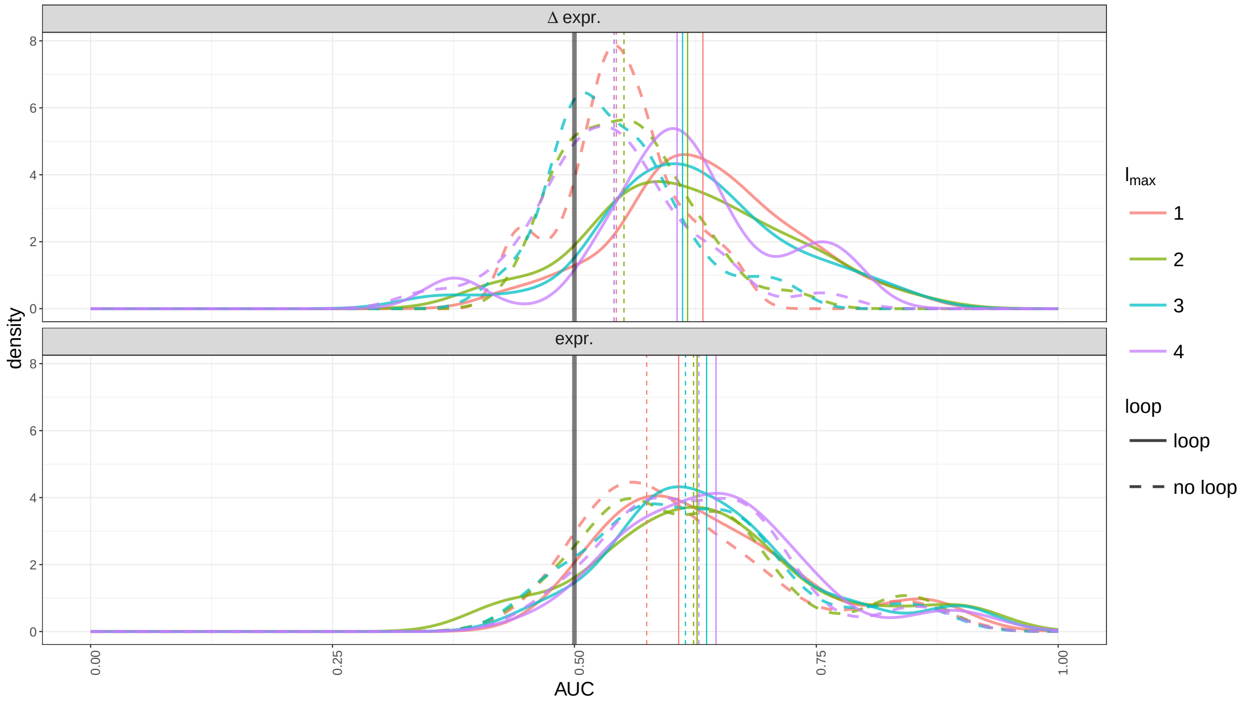

Figure S-5: Densities fit to the Ordered Lasso-based DREAM AUCs for each combination of model output, loop existence, and order $l_{\max}$. Vertical lines indicate empirical medians.

Several trends in the AUC can be observed with respect to the modeling parameters. For example, given a fixed $l_{\max}$ and loop inclusion choice, the AUC with the change in expression as the output tends to be less than the AUC with the expression as the output, suggesting that modeling the dynamics based on the change in expression may produce less accurate networks. In addition, the difference in the AUC appears to be larger in the non-loop case than in the loop case. Similarly, when $l_{\max}$ and the model output choice are fixed, excluding loops from the models results in less accurate networks, with the effect being greater in the change-in-expression output models than in the expression output models. Lastly, a variation of the monotonic AUC property that we observed when varying the model order in the HeLa subnetwork application can also be seen in the DREAM AUC densities. In this case, when the expression of a gene is used as the model output, the AUCs tend to increase with $l_{\max}$. However, when the change in expression is used, the AUCs now decrease with $l_{\max}$.

Altogether, these results impart several practical guidelines for predicting accurate networks with the Ordered Lasso. In general, using the expression to model the output tends to produce more accurate networks than using the change in expression. However, depending on the choice of the other parameters, the networks predicted by the latter approach may not be significantly different. Furthermore, using loops in the expression models to account for a gene's own variance in expression over time can also help the Ordered Lasso to predict accurate edges. Finally, the monotonic AUC property can be used to guide the choice of model order; if the expression (change in expression) is used as the output variable, then large (small) model orders should be used.

model	loop	$l_{\max}$	p.value
Δ expr.	loop	1	1.043081e-07
Δ expr.	loop	2	2.764165e-06
Δ expr.	loop	3	4.768372e-06
Δ expr.	loop	4	1.761317e-05
Δ expr.	no loop	1	1.693666e-04
Δ expr.	no loop	2	1.044422e-04
Δ expr.	no loop	3	1.022652e-03
Δ expr.	no loop	4	1.071442e-02
expr.	loop	1	2.235174e-08
expr.	loop	2	1.020730e-06
expr.	loop	3	5.215406e-08
expr.	loop	4	3.725290e-08
expr.	no loop	1	1.862645e-07
expr.	no loop	2	3.203750e-07
expr.	no loop	3	3.203750e-07
expr.	no loop	4	7.450581e-08

Table S-1: Wilcoxon test p-values for the Ordered Lasso-based DREAM AUCs at different model and parameter settings, with $H_0 : \mu_{\text{AUC}} \leq 0.5$ and $H_1 : \mu_{\text{AUC}} > 0.5$
